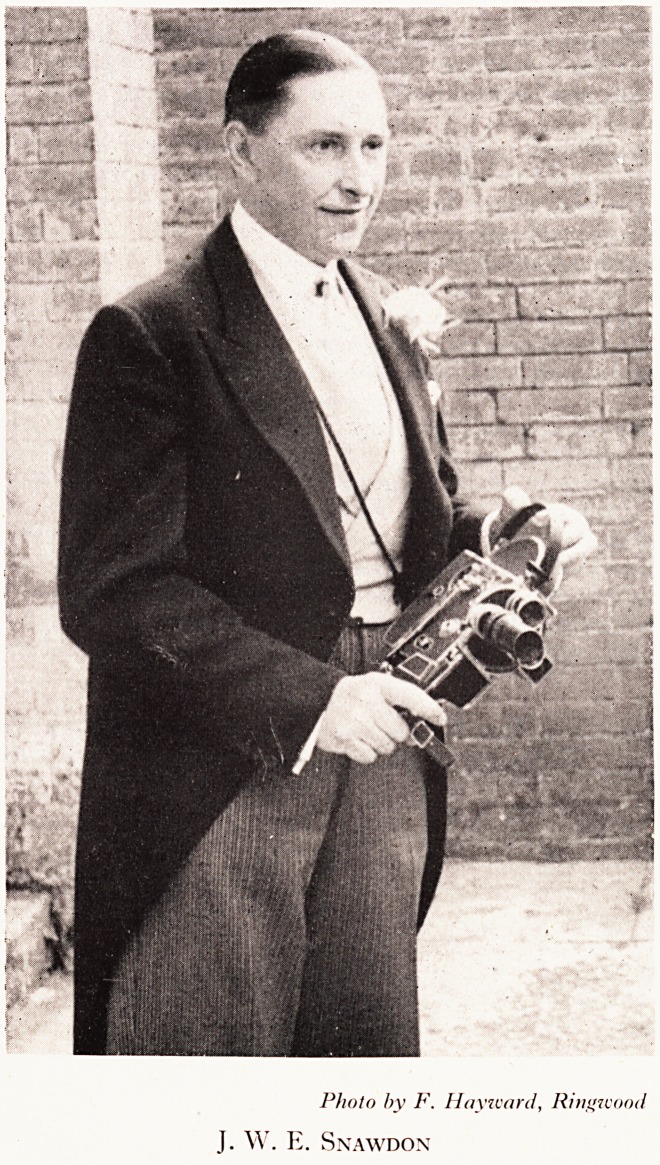# John William Edmund Snawdon

**Published:** 1962-01

**Authors:** 


*Photo by F. Hayward, Ringwood*

J. W. E. Snawdon

### OBITUARY

### JOHN WILLIAM EDMUND SNAWDON, M.D.S., M.B., Ch.B., F.D.S.R.C.S.

*13th February* 1912—*19th October* 1961

The passing of John Snawdon last month when still under fifty years of age was a tragedy felt very keenly in Bristol and among his many friends and colleagues in the South West.

John Snawdon went to the Bristol Grammar School and the University of Bristol, graduating in Dentistry with Honours in 1935. He continued his studies in Medicine to win the Gold Medal for his Year and graduate in Medicine in 1937. His aim was from the outset towards Oral Surgery and his training was continued in the Dental Hospital and in the Ear, Nose and Throat Department at the Bristol Royal Infirmary, with which latter work he was associated for several years. He commenced practice in Dental Surgery on his own in 1938.

The war supervened soon afterwards and he served in the Army Dental Corps in this country and in India, where he worked for three and a half years, for much of this time as a Specialist in Maxillo-Facial Dental Surgery, with No. 2 Indian Maxillo-Facial Surgical Unit.

On returning to civilian life he acquired higher degrees in Dental Surgery, both in Bristol and at the Royal College of Surgeons, and resumed his work as a Consultant Dental Surgeon at the Bristol Dental Hospital and Bristol Royal Hospital, Lecturer in the University of Bristol and Examiner in Bristol and later in Belfast. Within recent years he took on further work in Oral Surgery at Frenchay Hospital and in Consultant Dental Surgery at Weston-super-Mare.

For nine years he was Dental Assistant-Dean to the Faculty of Medicine of the University of Bristol, in which position he had an important influence on the Dental School and on all Dental Students for almost a decade. He was an excellent lecturer and devoted much time and effort to teaching.

He engaged in some clinical research and wrote a number of papers. In recent years he directed his activities more in the direction of oral surgery, having less time for dental practice. It is thought by many best qualified to know that he would have succeeded in forwarding this specialty in Bristol to the extent of becoming wholly occupied in oral surgery. His work was excellent and his services were becoming more and more in demand. He was a member of the Federation Dentaire Internationale and of the Oral Surgery Club.

In the administrative field he was singled out for considerable honour in becoming Chairman not only of the Dental Advisory Committee but also of the Medical Advisory Committee of the South Western Regional Hospital Board. His clarity of thought and methodical approach and transparent honesty fitted him admirably for this work.

Passing from his work to some of his other main interests, we must touch on cine-photography and sound recording, at which he was superlatively good. His films of operations were in wide demand, making him a very popular lecturer throughout the South-West and beyond. His talking film records in colour of Bristol during the Festival of Britain, of his visit to Rome and many others were a delight to those privileged to see them, whether in his home or in audiences to which he was always so willing to show his films. His technical ability in this sphere was very great and perfection as always his aim: he gave invaluable assistance to many of his colleagues at the hospitals in such matters. Other hobbies were stamp collecting, music appreciation and the growing of carnations.

Industry and a constant pursuit of perfection enabled him to make the most of his talents, which he freely offered to his fellow men and women. His calm serenity was greatly valued but with it all he had a nice sense of humour, to the service of which his pocket recorder had proved invaluable. He was fond of people and one of his greatest memorials will be the Annual Bristol Dental School Reunion, a gathering of old students for a day of clinical instruction and discussion, followed by a Dinner. This Reunion is the envy of many dental schools and was brought into being by John Snawdon.

His affection and loyalties were deep rooted, his convictions strongly held, yet he was not easily roused, holding annoyance and frustration which must go with strong feeling well in bound. He had a great facility for earning the loyalties of those with whom he worked and was held in affection and admiration by many. With all his gifts and accomplishments he was very modest.

He was a keen Christian, a Sidesman at his Church and a member of the Parochial Church Council at the time of his death. He leaves a widow and four young children, to whom we extend our sympathy in their great loss.

John Snawdon lived with much purpose and enthusiasm and with a certain dignity and charm which meant much to his friends, patients and associates. He will be greatly missed.

A. L. E-B.

## Figures and Tables

**Figure f1:**